# Cow's Milk and Dairy Consumption: Is There Now Consensus for Cardiometabolic Health?

**DOI:** 10.3389/fnut.2020.574725

**Published:** 2020-12-08

**Authors:** Sally D. Poppitt

**Affiliations:** Human Nutrition Unit, Department of Medicine, School of Biological Sciences, University of Auckland, Auckland, New Zealand

**Keywords:** fat, protein, dairy, CVD, diabetes, cardiometabolic

## Abstract

Cow's milk and dairy products derived from this complex food source have long been proposed as beneficial to human health, yet underlying clinical evidence of direct benefit continues to raise controversy. Limited evidence supports positive cardiometabolic effects of a number of dairy macro- and micronutrient components including whey protein and casein, unsaturated fats, milk fat globule membrane (MFGM) and polar phospholipids, vitamin D and calcium, in addition to non-bovine components including bacterial and yeast probiotics. More controversial remain lipid components *trans* fats, including *trans* vaccenic acid, *trans* palmitoleic acid, and conjugated *cis trans* linoleic acid (CLA), plus medium-chain and odd-chain dairy fats. New evidence is rapidly identifying multiple pathways by which these dairy nutrients may effect health. Processing, including fermentation and homogenization, may also have positive effects. Conversely, the high saturated fat content of dairy has long raised concern, aligned with international guidelines to minimize dietary intake of animal-origin saturated fatty acids (SFA) to achieve better cardiometabolic health. However, led in part by observational studies and meta-analyses showing dairy to have no or even an inverse association with cardiometabolic health, evidence from randomized controlled trials (RCTs) has been scrutinized over the last 5 years, and focus on low-fat dairy has been challenged. Recent evidence supports the hypothesis that adverse effects of SFAs on metabolic health may be ameliorated when these fats are consumed within a complex matrix such as milk, cheese or yogurt, and that dairy food categories may influence outcomes as much as total fat content. For example, yogurt and high-fat, high-SFA cheese have a negative association with risk of type 2 diabetes (T2D) in many, not all, published trials. However, large sample dairy RCTs of long duration with CVD or T2D incidence as primary endpoints are lacking. This is a clear research gap, with these clinical studies required if a causative link between dairy and improved cardiometabolic health is to be confirmed and in turn promoted through dietary guidelines. Current advisories from national guidance groups such as American Heart Association (AHA) and European Society of Cardiology (ESC) continue to promote consumption of low-fat dairy products, whilst liquid milk and yogurt remain part of nutrition guidelines from joint American Diabetes Association (ADA)/European Association for Study of Diabetes (EASD) reports, and as part of a “no-one-size-fits-all” answer to diet and T2D by the ADA in their most recent 2019 Consensus Report.

## Introduction

Cow's milk and dairy products derived from this complex food source have long been proposed as beneficial to human health, yet underlying clinical evidence supporting direct benefit to cardiometabolic health continues to raise controversy, based primarily on the high saturated fatty acid (SFA) content of whole-fat dairy. Whether high levels of dietary SFAs *per se* do indeed initiate a cascade of worsening intermediary blood markers including an adverse lipoprotein profile, and in turn lead to higher prevalence of cardiometabolic disease is now under considerable scrutiny. This narrative review aims to present the most recent evidence from both observational cohorts and randomized controlled trials (RCTs) that investigate the relationships between dairy and risk of cardiovascular disease (CVD) and type 2 diabetes (T2D), and to evaluate the evidence provided by these different study types. Also to identify, where possible, potential mechanisms by which dairy nutrients may promote health benefits.

Concern around dairy and potential adverse health outcomes arose from early epidemiologic data which supported a strong association between animal-origin food groups that provide a major source of dietary SFAs and an increased risk of CVD (1). In countries such as the UK, milk and dairy foods contribute almost 30% of SFA intake, and so quite reasonably have been considered a food component of concern (2). However, current literature shows growing support for the proposal that dairy products may have a neutral or even positive effect on CVD outcomes (3), with a number of meta-analyses supporting this relationship (4–9). There is also a new but growing consensus that the matrix of a whole-fat food such as dairy may be more important than the content and composition of component isolated fatty acids. Such that food-based rather than nutrient-based recommendations should be developed for CV health (3, 10, 11). It is notable however that the predominance of this evidence is obtained from observational studies. There is less evidence from RCTs, particularly with reference to incident CVD where long-term dairy interventions evaluating hard CV event points are lacking. This may be critical when aiming to interpret findings suggestive of a positive relationship with regular- and high-fat diary, and in order to develop robust public health recommendations.

A number of meta-analyses and systematic reviews have also focused on dairy and T2D (4, 12–17), again showing an inverse association between dairy intake and risk of T2D in observational studies. A recent expert panel position paper (18) reported a number of key findings including the evidence from large prospective cohort studies that total dairy consumption has a neutral or moderately beneficial effect on T2D risk. Again notably this is an outcome supported only by limited evidence from randomized controlled trials (RCTs) (19), with no long-term interventions investigating the effect of high-fat dairy on incident T2D. Dairy category is clearly important, again with evidence from prospective cohort studies showing fermented yogurt to be most strongly associated with lower T2D risk. There is with sparse evidence from RCTs. Even in 2020, the balance of evidence is predominantly from observational studies. Larger and longer duration clinical intervention trials are needed, with incident T2D as the primary outcome. RCTS are also required for better understanding of the underpinning mechanisms by which dairy may potentially be protective.

Dairy composes about 10% of the energy consumed in, for example, a typical North American diet, of which approximately half is from fluid milk, half from fermented (or “cultured”) cheese, and a small percentage from fermented yogurt (20). Important nutrients found in the myriad of dairy formats include milk proteins, calcium, magnesium, potassium, medium- and odd-chain saturated fats, specific fatty acids, and low-glycemic index (GI) sugars; shown to have beneficial effects on aspects of glucose control, insulin secretion, insulin sensitivity and/or T2D risk (21) as well as a range of CV risk factors (22). Notably a number of authors (23–26) have recently highlighted the importance of focusing on foods and dietary patterns rather than simply individual dietary nutrients when assessing CVD and T2D risk. In turn it is clear that expanding response to these dietary patterns beyond simple body fatness and circulating blood lipids into the multiple risk outcomes now identified as important to CV health is important.

## Methods

A literature search was conducted using MEDLINE (via PubMed) to identify observation cohorts and intervention trials that investigated the association between dairy consumption and cardiometabolic health. Medical Subjects Headings (MeSH) included dairy OR milk OR butter OR cheese OR yogurt AND CVD OR diabetes; reviews, including systematic reviews, meta-analyses, umbrella reviews, narrative reviews.

## Cardiometabolic Effects of Dairy Macro- and Micro-Nutrients: Evidence From Clinical Studies

### Dairy Proteins

#### Whey and Casein

Among the various types of animal-based protein foods, a higher intake of protein-rich dairy products has been reported to be associated with a beneficial relationship with a range of CV endpoints in addition to glucose regulation and T2D risk reduction (16, 19, 27, 28). Milk proteins can be categorized in the simplest terms into two groups, based on solubility, as serum or whey proteins and caseins. The whey proteins remain soluble at pH 4.6 and 20°C, whilst the caseins (or “curds”) precipitate out. Molecular and physicochemical properties of whey protein and caseins are highly varied, with the ratio of total whey:total casein in cow's milk of ~20:80% (29).

Although a number of large observational cohort studies do support the association between higher intake of dairy foods and neutral or lower risk of adverse CV health, data is sparse from observational studies for whey or casein *per se*. There however are several systematic reviews of RCTs that identify positive effects of whey protein. Wirunsawanya et al., recently reported on whey supplementation and CV endpoints in 9 RCTs of overweight and obese cohorts, and identified an improvement in serum lipoproteins for total and HDL-cholesterol in addition to improvement in blood pressure (30), although notably these effects were likely driven by body weight loss. There has been a growing literature on positive effects of whey protein on hypertension, where mechanisms are purported to include angiotensin converting enzyme (ACE) inhibition, normalization of endothelial function and opioid receptor-dependent effects, although not all studies show positive long-term outcomes (31). Recently a comprehensive systematic review by Badely et al. >2,000 individuals from 37 published RCTS, again in overweight and obese adults, showed whey protein administered in multiple forms including protein isolate, concentrate, extract, supplement and hydrolysate had a positive outcome on several CV markers. They reported a decrease in fasting triglycerides and blood pressure, but also an adverse decrease in HDL-cholesterol (32). Whilst not reporting change in body weight these outcomes were accompanied by a decrease in waist circumference, which is an indirect measure of central adiposity expected to also be accompanied by weight loss. Whey protein has long been shown to have modest positive effects on body composition during weight loss (33), but whether there are effects of whey protein on CV endpoints independent of change of body weight remains to be convincingly demonstrated. Previous observational studies have reported weight independent effects of total protein intake on cardiometabolic health, with authors in turn noting that the role of protein in CV health likely depends on the specific protein source (34).

Evidence that protein-rich dairy products may be beneficial for T2D has largely come from observational studies (27) where again attribution of positive effects of metabolic health to dairy proteins *per se* is equivocal. Notably the relationship between increased intake of total protein from the diet and cardiometabolic endpoints is not without controversy (35, 36), in particular for T2D where some cohort studies show high total protein or animal protein intake to be associated with an increased risk of disease (37–39), including some proteins of dairy origin (40). Clearly protein source and quality is of considerable importance when determining dietary guidelines for prevention of T2D. Clinical evidence from RCTs regarding both dairy foods and dairy proteins point to wide ranging interventions reporting enhanced insulin secretion and associated endpoints, which in turn may result in better glycemic control. Wirunsawanya and colleagues, in their meta-analysis of whey protein from nine RCTs in overweight and obese referred to previously, showed significant improvement in fasting glucose albeit alongside a parallel decrease in body weight (30). Badely et al., also reported improvements in blood glucose in their systematic review of 37 published whey protein intervention trials in overweight and obese (32), but again whether this is independent of weight loss is not determined. This body of evidence is predominantly based on short duration studies of intermediary metabolic endpoints rather than long-term trials of incident T2D prevention, with long-term interventions of dairy protein on T2D endpoints or incidence lacking. Two trials planning to investigate moderate-term whey protein intervention for T2D management over a 3 months duration can be identified through review of international clinical trial registries. Almario et al. who have published acute effects (41) were unable to undertake their trial due to personnel changes [information kindly provided by the investigators; (42)]. Jakubowicz et al. (43) showed decrease in both postprandial glucose and HbA_1c_ on the high whey protein arm when compared with a low protein soy control. Notably, long-term dietary interventions for T2D prevention in overweight and high risk individuals to date are focused almost entirely on manipulation of the fat and carbohydrate components of the diet (44). The large European-led 2,500 participant, 3 years RCT, PREVIEW (PREVention of diabetes through lifestyle intervention In Europe and around the World) is the first long-term study to compare a novel higher total protein diet with a higher carbohydrate diet for incident T2D prevention (45). No significant improvement over the current best practice higher carbohydrate diet was found however. No long-term dairy protein RCTs investigating prevention of incident T2D have as yet been reported.

**Figure 1:**
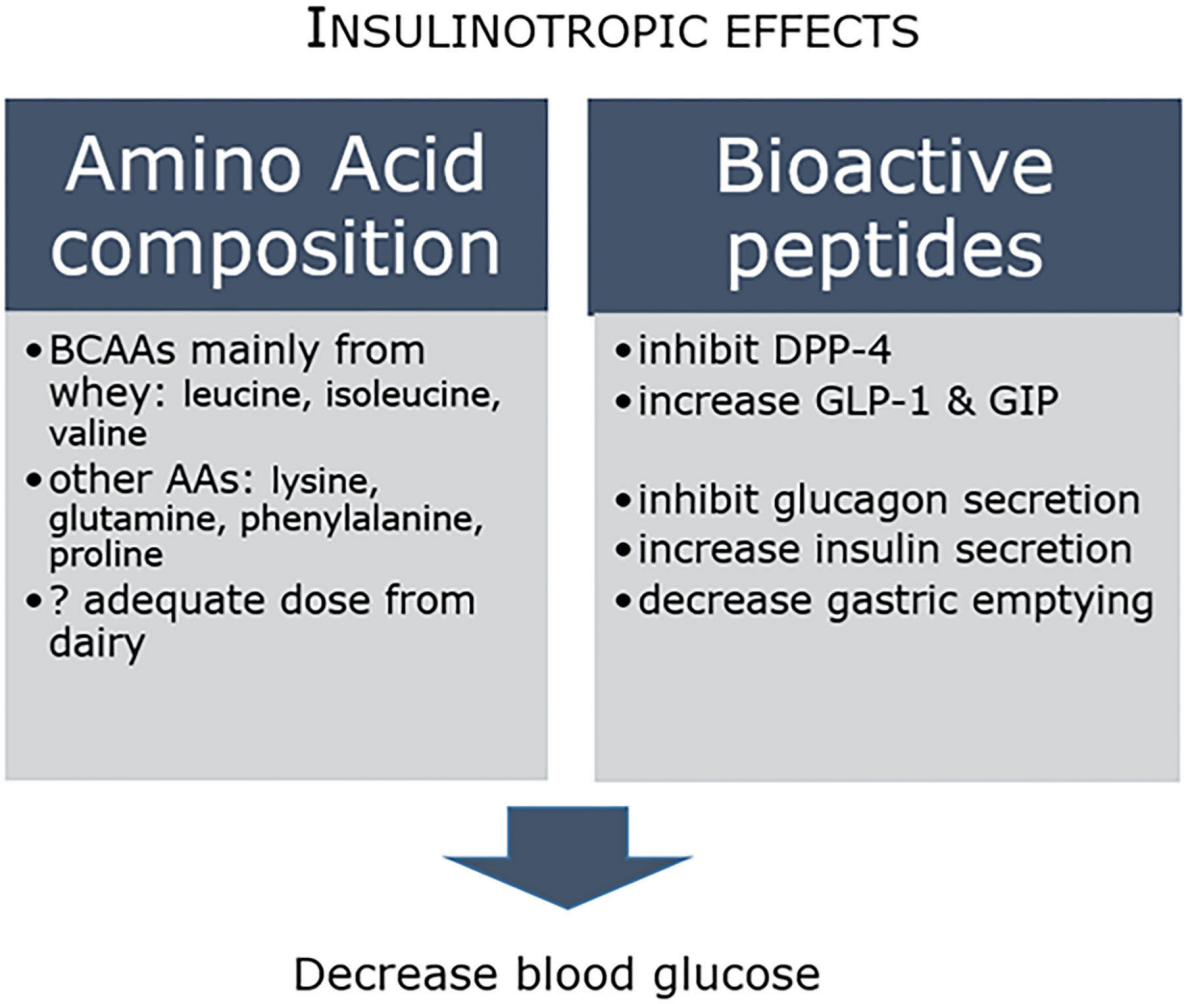
Potential mechanisms of glucose lowering by dairy caseins and whey proteins.

Acute intervention studies have provided some insights into potential mechanisms (46), with evidence that dairy proteins have more potent effects on insulin and incretin secretion compared to other commonly consumed animal proteins (21). Insulinotropic effects are associated with both the amino acid composition and the bioactive peptide profile, where for example inhibition of dipeptidyl peptidase-4 (DPP-4) increases incretin levels, which in turn inhibits glucagon release, increases insulin secretion and decreases gastric emptying (Figure 1). In concert, these in turn decrease circulating blood glucose concentrations. The amino acid content is driven in large part by the major contribution of whey protein to dairy, and consequent high levels of branched-chain amino acids (BCAAs) leucine, isoleucine and valine, as well as lysine (47). Clinical studies have reported dairy AAs, including leucine, isoleucine, glutamine, phenylalanine, proline and lysine, to have beneficial effects on glucose homeostasis. BCAAs stimulate the secretion of insulin and glucagon in addition to incretins glucagon-like peptide-1 (GLP-1) and glucose-dependent insulinotropic polypeptide (GIP) (47). In practical terms however, whether the dose of amino acids required to attain these protective responses can readily be achieved through recommended daily dairy intake is less well established (48). Clearly, more longer-term studies that can unravel the relationship between dairy protein and insulin and glucose control are needed.

In summary, there are no large RCTs evaluating effects of long-term dairy protein intake on either CVD or T2D incidence. There are however some preliminary conclusions to be drawn. Whilst cohort studies relating whey and casein *per se* to CV and T2D outcomes are lacking, RCTs have identified beneficial moderate-term effects of whey protein on intermediary risk factors including lipids and lipoproteins, blood pressure, and glucose-related parameters including insulin secretion. Acute postprandial studies provide evidence for whey protein and constituent AAs as promoting insulin secretion as a mechanism for improved glycemic control. Since much of the RCT evidence, however, has been obtained in overweight cohorts, the possibility that body weight and adipose mass loss may contribute to improvements in metabolic health seen in these dairy intervention trials cannot be excluded.

### Dairy Fats

The recent expert panel position paper referred to previously also reported on evidence underpinning relationships between dairy fatty acids and metabolic health. The panel concluded that medium-chain fatty acids/triglycerides (MCFA/MCT), odd chain fats, very long-chain SFAs and *trans*-palmitoleic acid were associated with lower T2D risk and improved metabolic health (18). They also noted, however, the possibility that findings were confounded by dairy fatty acids acting as markers of overall dairy consumption. Other evidence has been presented for milk fat globule membrane (MFGM) and polar phospholipids, *trans* vaccenic acid, and conjugated linoleic acid (CLA) in relation to both T2D and CVD risk, as summarized below.

#### Medium Chain Triglycerides (MCT)

MCTs are classified as lipids containing medium chain fatty acids of 6–12 carbon chain length. They are minor components of global diets but have long been investigated as a potential dietary lipid substitute for the more abundant long chain triglycerides (LCT). Milk is a reasonable source of saturated MCTs, comprising up to ~15% of total lipid (22). MCTs undergo rapid hydrolysis and absorption, and suppress lipedema, hypothesized to predispose MCTs as cardioprotective (49). Evidence remains preliminary however. MCTs undergo almost complete hydrolysis to free fatty acids (FFA) and are absorbed directly into the portal vein, and hence transported rapidly to the liver for oxidation. This is in contrast to LCTs which are more slowly absorbed and transported by chylomicrons into the systemic circulation prior to oxidation or storage. The concept of rapid oxidation enhancing peripheral satiety signals and in turn promoting body weight control has been investigated in several studies, but with little underpinning evidence from human studies (50).

Evidence of enhanced metabolic health comes from several sources. *In vitro* studies of skeletal muscle cells show MCTs to enhance oxidative mitochondrial capacity and decrease lipid accumulation relative to LCTs, but not activate NF-κB or decrease insulin sensitivity (IS) (51, 52). Preliminary evidence also exists from *in vivo* studies, reporting ameliorated body fat accumulation (53) and insulin resistance in animals fed MCTs vs. saturated LCTs, which aligns with these prior studies. In the DairyHealth study, a 12 weeks cow's milk intervention conducted in Europe, dairy MCTs have been shown to induce gene expression of energy metabolism-related pathways in adipose tissue samples collected by biopsy from adults with abdominal obesity (54). These authors also showed a cardiometabolic-protective decrease in inflammation-related gene expression. Conversely, in animal models MCTs have also been shown to increase hepatic *de novo* lipogenesis and triglyceride accumulation, and adversely decrease hepatic IS, when compared with a lower fat diet (51, 53). Ameliorated lipid storage has also been reported in clinical studies of overweight adults, including following consumption of high-MCT dairy butter in combination with dairy protein (55). The DairyHealth study of abdominally obese adults also reported a higher intake of milk MCT to increase lean body mass and decrease total body fat % over 12 weeks, in an isoenergetic fat quality manipulation (56).

#### Odd Chain

The odd-chain fatty acids (OCFAs), pentadecanoic (C15:0) and heptadecanoic acid (C17:0), are SFAs that are found in dairy. Minor components that comprise ~1.5% of milk fat, with C15:0 reported as approximately twice as abundant as C17:0. Long investigated as potential independent serum biomarkers of dairy intake (57, 58), these SFAs with odd number of carbons were proposed to be synthesized only by bacterial flora of ruminants (59, 60) with no metabolic precursors in humans. However, this has been challenged by emerging data of endogenous synthesis and metabolism of these OCFAs (61, 62). Adipose tissue depots rather than circulating fatty acids have more recently shown promise, with evidence that adipose C15:0 may reflect both habitual intake as well as changes in intake of dairy foods (63). With respect to health, recent reviews have reported that dairy OCFAs may be inversely associated both with cardiometabolic risk (20) and T2D (63) in cohort studies. C15:0 has also been proposed as an essential fatty acid (64) with some evidence in animal models of amelioration of inflammation and dyslipidemia.

#### Milk Fat Globule Membrane (MFGM) and Polar Phospholipids

The MFGM is the biological membrane that surrounds the lipid droplets in liquid milk. This and associated phospholipid (PL) components of milk continue to be associated with health benefits including those of metabolic health, as recently reviewed by Anto et al. (65) in a narrative review which presented 11 RCTs of milk PL effects on circulating serum lipids. A growing evidence base is proposed for conditions as varied as adverse lipid metabolism (66–68), insulin resistance (69), inflammation (70), CVD through attenuated development of atherosclerosis (65), gut health (71) and neurodevelopment. Structurally the MFGM encases the fat globules within milk. It is comprised of proteins, cholesterol and polar rather than neutral lipids, including PLs such as phosphatidylcholine (PC) and sphingolipids such as sphingomyelin (SM). Recent mass spectral analysis of bovine milk has confirmed PC, phosphatidylethanolamine (PE) and SM as the most abundant polar lipid classes (72). The total polar lipid content of a product may vary greatly as a result of dairy processing, but typically comprise only ~1% of the total lipid content of milk. Yet they contribute to several classical dairy food performance characteristics. The ability to stabilize oil-in-water emulsions is one of these, such that the polar lipids enable the emulsification of neutral triglyceride in the aqueous phase of liquid milk. When consumed in significant amounts within the diet, PLs can inhibit lipid absorption from the GI tract, acting mainly by altering mobilization of lipid from spherical micelles that form in aqueous solution (73). Disruption of the MFGM, such as during homogenization, is discussed in more detail in sections below.

#### *Trans*-vaccenic Acid and *Trans*-palmitoleic Acid

**Figure 2:**
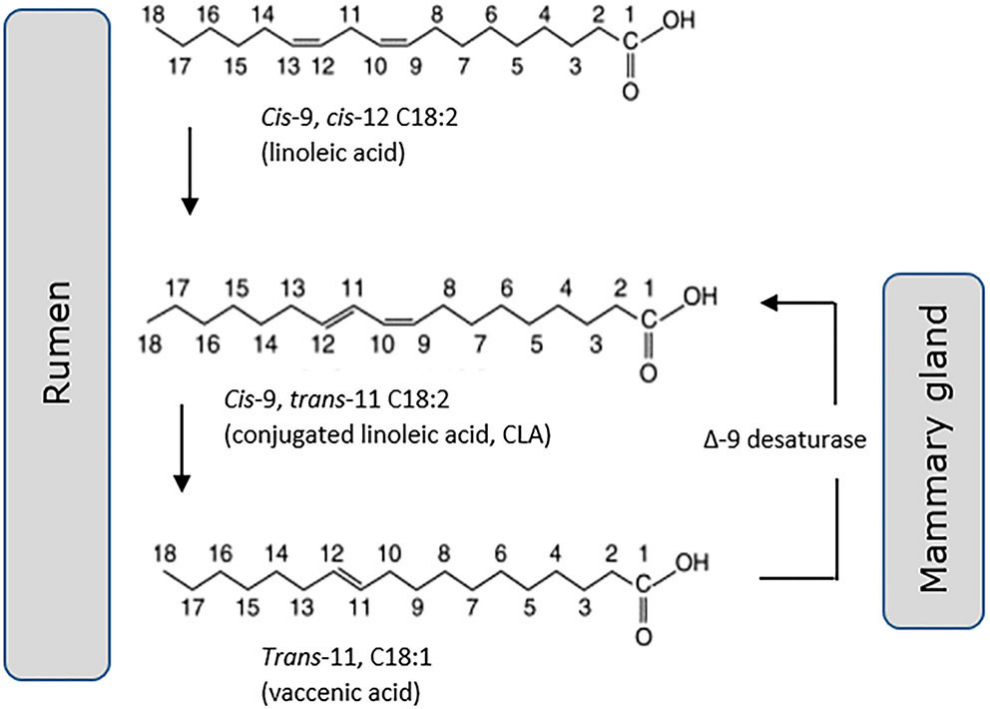
*Cis*-9, *trans*-11 CLA (18:2) is generated both through hydrogenation of linoleic acid (18:2) in the rumen and desaturation of *trans*-11 vaccenic acid (18:1) in the mammary gland.

*Trans*-11 vaccenic acid (C18:1, t11) is a monounsaturated “natural” *trans* fat, specific to ruminant-origin products of dairy and meat. There is some evidence (74), although not universal (75, 76), that it lacks the adverse cardiometabolic health outcomes associated with other forms of dietary *trans* fats, long developed by the food industry through industrial lipid hydrogenation. There are a number of *in vivo* and *in vitro* reports of positive effects of dairy *trans* fats on metabolic endpoints including increased insulin secretion and pancreatic islet β-cell growth (77), decreased hyperlipidemia (78), and ectopic liver fat accumulation (79). These reports however must be balanced against others that do not support a health benefit. A 2010 systematic review of 39 clinical intervention trials by Brouwer et al. (75) included 29 industrial *trans* fats, 17 CLA and six ruminant *trans* fats studies. They concluded that all fatty acids with a *trans* double bond raised the LDL- to HDL-cholesterol ratio in an adverse manner. In an update of this review they noted the contradictory evidence that observational studies fail to demonstrate a higher risk of CVD associated with high intake of ruminant *trans* fats, despite confirming adverse effects on circulating blood lipid profile (80). Conversely, evidence of positive outcomes for T2D has been building, where monounsaturated *trans*-palmitoleic acid (16:1, t9) is of particular interest. Associated in recent publications with a lower incidence of T2D (18, 81) and coronary artery disease (CAD) (81), it is both consumed within dairy as well as delivered through endogenous conversion from its metabolic precursor *trans*-vaccenic acid (82). Evidence for a protective effect of dairy *trans*-palmitoleic comes from a recent large meta-analysis of 16 prospective cohort studies comprising more than 60,000 participants. Conducted by the fatty acids and outcomes research consortium (FORCE) this analysis showed a significant association of *trans*-palmitoleic with a lower risk of T2D (58). A note of caution must be added however, with several authors questioning the robust nature of methods used for analysis of fatty acid methyl esters (FAMEs) from bovine milk, citing difficulties in full separation and unequivocal identification of FAMEs within the very complex lipid composition of ruminant milk (83, 84). The various pathways linking these *trans* fats to CLA are now reasonably well understood, including desaturation of *trans*-11 vaccenic acid (18:1) by the Δ9-desaturase enzyme within the mammary gland to form *cis*-9, *trans*-11 CLA (18:2) (Figure 2). This is in addition to formation of CLA via biohydrogenation of linoleic acid (18:2) within the bovine rumen, and in turn synthesis of *trans*-vaccenic. Synthesis via Δ9-desaturase has been proposed to be the primary source of *cis*-9, *trans*-11 CLA in bovine milk fat (85).

#### Conjugated Linoleic Acid (CLA)

CLA is a polyunsaturated fatty acid (PUFA) with conjugated double bonds found in both *cis* and *trans* forms, the most common isomer of which is *cis*-9 *trans*-11 linoleic acid. Despite being found in ruminant dairy and meat, and in lesser amounts in plant oils and seafood, CLA is also widely produced industrially for sale as a supplement. Much like vaccenic acid, there are reports of cardiometabolic health benefits (86, 87). A systematic review and meta-analysis of 33 RCTs that investigated the effect of ruminant CLA, in either supplement form or from enriched foods, on lipid profile in healthy adult populations reported that both formats significantly decreased LDL-C with minor non-significant effects on other lipid outcomes (87). The review also however noted concerns on potential safety of CLA with reports of increased insulin resistance and lipodystrophy in animal models, in addition to some adverse lipid and C-RP responses in clinical studies. In line with this note of caution, several recent reviews have concluded that many positive outcomes reported in animal models or *in vitro* have not been adequately replicated in clinical trials (88, 89). The 2018 review by Li et al. reported the lack of clinical data for hard CV endpoints. They identified only 1 observational case-control study showing an inverse relationship between levels of cis-9, *trans*-11 CLA in adipose tissue and the risk of heart attack (90), and 3 RCTs all showing no significant effect of CLA on blood lipids, glucose, CRP, blood pressure, insulin resistance, body composition or 10-year absolute risk of fatal CVD in obese patients (91). There was a similar lack of efficacy reported in healthy cohorts (92) and patients with atherosclerosis (93). There is evidence of improved C-RP levels from one intervention trial in patients with active CVD (94). Conversely there is evidence, as for vaccinic acid, that CLA adversely affects lipoprotein profile, with increased circulating LDL-cholesterol and total:HDL-cholesterol ratio in an exhaustive review of 48 CLA and 11 ruminant *trans* fat RCTs (80). In addition, worsening of associated CVD risk factors including the acute phase protein C-RP [Haghighatdoost and Nobakht-M-Gh 2018, (95)] and cytokine TNF-α have been identified in a recently published systematic review (96). In addition, a 2017 meta-analysis of 32 RCTs of food-enriched and supplement CLA has also shown no significant effect on fasting glucose (88). A recent narrative review has noted lack of consensus interpreting effects of CLA on body weight loss and obesity due to small sample size, variable dose, variable CLA isomers, variable intervention durations and population characteristics (97); but that beneficial effects of supplementation on parameters related to body weight and/or adiposity have been reported in a number of trials, with no evidence of adverse metabolic consequences. This review also reports similar absence of consensus for glycemic endpoints, with both beneficial glucose metabolic effects and no clinical benefit reported in RCTs (97).

In summary, there are no RCTs evaluating long-term effects of these specific dairy fat components on either CVD or T2D incidence. Very little evidence supports MCTs as cardioprotective, with preliminary clinical findings related only to modest improvements in body composition. In turn, OCFAs have been associated with decreased CV and T2D risk in cohort studies, but no intervention data is available. Conversely, there is limited evidence that MFGM and the associated PL components of dairy may improve CV and T2D intermediary endpoints, in RCTs of 1 day to 12 weeks duration reporting outcomes including lipid metabolism, inflammatory response and insulin resistance. The evidence that underpins *trans-*fats is more complex and controversial. Ruminent-origin *trans* vaccenic acid (*trans*-VA) may not generate the well-demonstrated adverse response in CV risk endpoints of industrial *trans-*fats. There is lack of concordance in the evidence base however, with proposed neutral effects of *trans*-VA observed in cohort studies not being supported by RCT evidence, where systematic review has concluded that all fatty acids containing a *trans* double bond adversely increase lipoprotein profiles, irrespective of their origin. Conversely there are reported positive T2D risk outcomes for the downstream product of *trans*-VA, *trans*-palmitoleic acid (*trans*-PA) which is also present in minor quantities in dairy, and which cohort studies have correlated with decreased T2D risk. CLA has also been proposed as cardioprotective, with both ruminant-origin and industrial-origin supplements found within the western diet, and highly variable levels of evidence published. From meta-analysis of RCTs showing both significant improvement and significant worsening in lipoprotein risk factors, alongside no significant effects on FPG, to very limited data supporting an association with improved CV incidence in observational studies. There remains no current consensus on outcomes likely due to factors including the highly variable isomers and dose of CLA consumed, size and type of experimental cohorts recruited, and duration of the RCTs.

### Micronutrients: Vitamin D, Calcium

Relationships between vitamin D, calcium, and various aspects of cardiometabolic health have long been controversial. In this review, focus has been given to the evidence underpinning relationships between vitamin D and T2D, and calcium and CVD. Dairy naturally contains little vitamin D but is a food category much debated with respect to fortification. Fluid milk and its myriad products have a history of both mandatory or voluntary fortification with vitamin D by the food industry. A recent review reported milk products currently to be systematically, either mandatory or voluntary, fortified with vitamin D only in European Nordic countries of Finland, Norway, Sweden as well as in North American countries of Canada and the United States (98).

**Figure 3:**
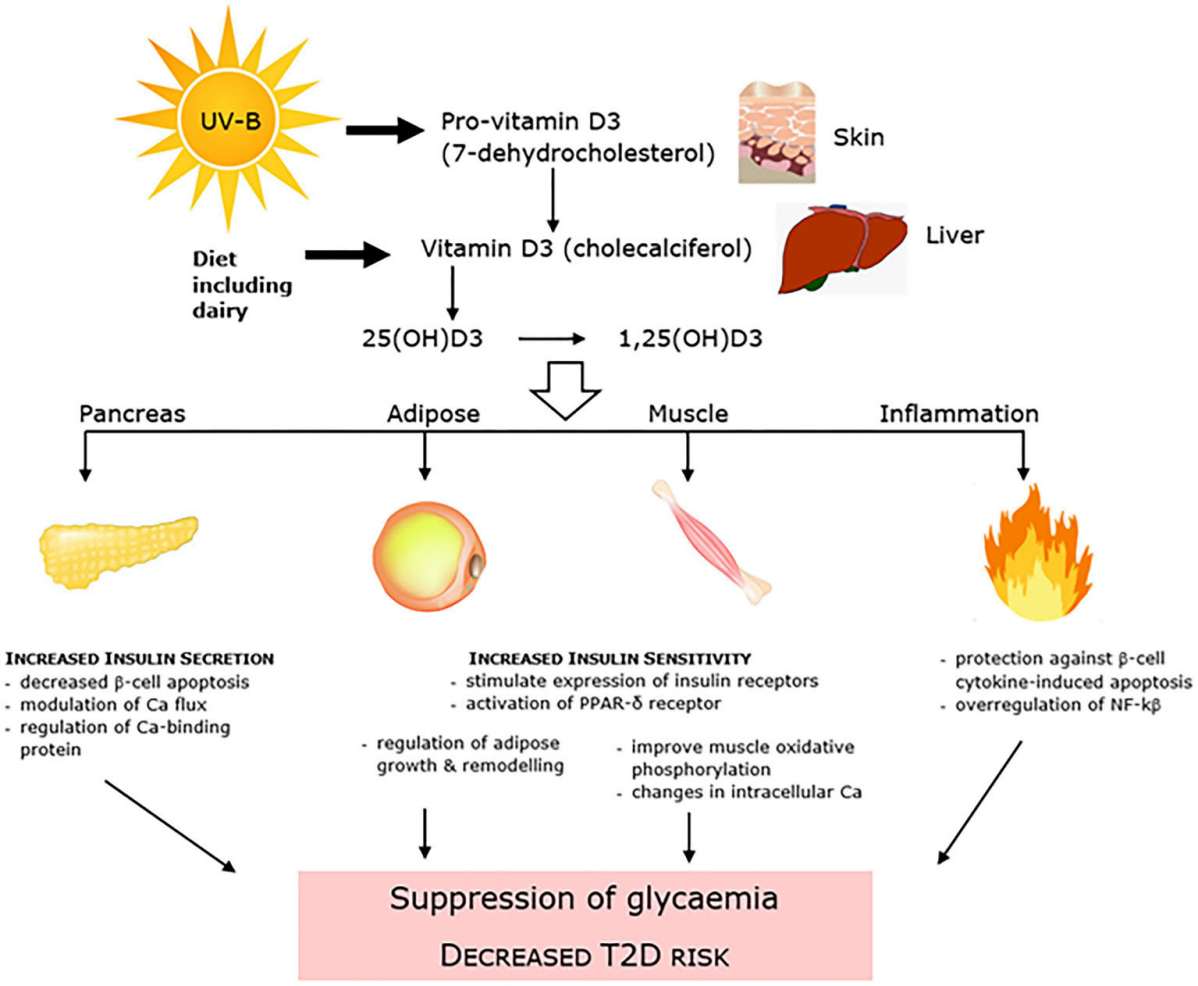
Vitamin D is linked to dysglycaemia and T2D through mechanisms that affect both pancreatic β-cell insulin secretion and insulin resistance, as well as systemic inflammation.

There has been conflicting observational evidence for a number of years which has linked low levels of 25-hydroxyvitamin D (25(OH)D) to T2D, and has led to vitamin D replacement interventions conducted with intent to both improve glycemic control and decrease T2D incidence (99). Both synthesized in the skin in response to sunlight exposure and/or consumed within the diet and as a supplement, 25(OH)D is the main circulating form of vitamin D. It has been linked to dysglycaemia and T2D through mechanisms that affect both insulin resistance and pancreatic β-cell insulin secretion, as well as inflammation and accumulation of advanced glycation end (AGE) products (Figure 3). A number of early systematic reviews and meta-analyses supported the protective inverse relationship between vitamin D and T2D. An analysis of 8 observational cohort studies reported that high vitamin D status (>25 ng/ml) was associated with an almost halving of risk of T2D compared with low status (100). Another more recent review of 21 cohort studies showed a similar relative risk of 0.62 and using linear trend analysis also reported that for each 10 nmol/L increment in circulating 25(OH)D levels there was an associated 4% lower risk of T2DM (101). A further meta-analysis of 16 cohort studies also showed a positive outcome (102). Adiposity and adipose mass is a confounding factor in the relationship between vitamin D and T2D. Vitamin D is sequestered in adipose tissue, with the greater adipose mass reported to act as a “reservoir” for vitamin D, and the increased amount of vitamin D required to saturate the large depots of obese individuals predisposing to low levels of circulating 25(OH)D (103). In turn obesity has long been identified as a very strong predisposing factor for T2D (104).

Far fewer clinical intervention trials have been conducted, with confounding issues including failure to reach sufficient circulating levels of vitamin D (105). Required circulating levels of 25(OH)D necessary to effect glycemic control and decrease risk of developing T2D, have not yet been determined. It is speculated that these may be higher than that required for optimal bone health (99). Conclusions have varied widely from meta-analyses that report positive cardiometabolic outcomes, including findings from an analysis of 9 RCTs which showed that vitamin D-fortified yogurt decreased HOMA-IR, fasting serum glucose and total cholesterol in normoglycaemic and T2D patients (106), and from analysis of 19 RCTs which showed improvements in both IR and HbA_1c_ in T2D patients (107). Conversely prior systematic reviews reported no effect of vitamin D supplementation on glycemic outcomes in a meta-analysis of 11 (100), and 15 RCTs of normoglycaemic and T2D patients. The latter however showing that sub-cohort analysis of T2D or IGT patients resulted in a small effect on fasting glucose and IR, but not HbA_1c_ (108).

Calcium and metabolic health has been under considerable scrutiny in recent years. Overall, calcium is not a convincing contributor to cardiometabolic benefits that may be seen from dairy. Commonly used as a supplement to promote bone health with contributions to prevention of fracture risk in the elderly, meta-analysis of long-term RCTs unexpectedly identified calcium supplementation to result in moderately increased risk of heart attack (109, 110). The mechanism that may underpin this risk has been proposed to be postprandial hypercalcemia, in turn contributing to vascular calcification. Interestingly, single-nucleotide polymorphisms (SNPs) related to higher serum calcium levels have more recently been associated with increased risk of coronary artery disease and myocardial infarction in genome wide studies (111). It has become clear, however, that it is important to differentiate the origin of the calcium consumed, with different effects between calcium consumed as a dietary supplement vs. calcium consumed as a food from dairy sources. Calcium supplements, but not calcium consumed within food products, have been proposed to result in a negative risk-benefit, with routine use for prevention or treatment of osteoporosis no longer universally recommended (112). Indeed, current evidence no longer supports routine calcium or vitamin D supplementation for bone health in healthy adults resident within the community, based in major part on the adverse CVD outcomes (113).

The evidence in support of dairy-origin calcium for T2D prevention is also mixed, although as recently reviewed (22), there are multiple mechanisms by which calcium may influence T2D risk factors. This includes regulation of insulin-mediated intracellular processes in specific tissues that respond to this peptide, contribution to secretory function of β-cells within the pancreas, and phosphorylation of insulin receptors. Calcium has also been shown to down-regulate genes encoding pro-in?ammatory cytokines that are involved in IR. In their review Mozaffarian et al. also reported >20 observational studies on associations between calcium intake and T2D prevention (22), of which 3 large cohort studies in US and China showed an inverse association between high calcium intake and low T2D risk in subcohorts of women but not men. A 4th large cohort study in Japan also observed this inverse relationship but only in subcohorts with higher serum vitamin D. The other smaller studies reported mixed outcomes, with no clear consensus. Additional to this review, the Korean Genome and Epidemiology study [KoGES (114)], a prospective cohort trial followed for 10 years, reported the positive outcome that higher dietary calcium, but not serum calcium, was associated with a lower risk of developing T2D in a study of >10,000 adults from an Asian cohort.

### Processing: Fermentation, Probiotics, and Homogenization

There is considerable evidence that processing methods including fermentation, addition of probiotic bacteria and yeasts, and homogenization can all influence cardiometabolic health, as presented in the sections below.

#### Fermentation

Both cheese (“cultured”) and yogurt are commonly consumed fermented dairy products. The fermentation process requires a starter culture, which may vary greatly in order to achieve required texture, flavor and safety profile. Lactic acid bacteria are the major bacteria used in the fermentation process of modern food products, and can include the genera *Lactobacillus, Lactococcus, Streptococcus, Leuconostoc, Pediococcus*, and *Enterococcus*, whilst yeasts and molds are also used in cheese production (115). Acidification rate through production of lactic acid in addition to the secretion of secondary metabolites, including bacteriocins, biogenic amines, exopolysaccharides, and proteolytically released peptides, are key aspects of the fermentation process. Notably, neither cheese nor yogurt exert the adverse effects on blood lipids that would be predicted solely by the content and composition of their component SFAs (116, 117). Consumption of yogurt is clearly associated with decreased risk of both T2D and cardiometabolic disease, as reviewed by the recent expert panel of Guo et al. (18). Whilst a number of factors may contribute, the fermentation processes and the probiotic content and composition have both been proposed as significant contributors.

#### Probiotics

Probiotic bacteria are common components of fermented foods, including dairy set and drinking yogurts and the fermented dairy drink kefir. Kefir, similar to a liquid drinking yogurt, is a more recent introduction to the wider Westernized diet, prepared through inoculation of ruminant milk with kefir “grains,” a product which contains both bacteria and yeasts. Multiple probiotic genera have been evaluated in cohort studies and RCTs, with the two most common being *Lactobacillus* and *Bifidobacterium*. Probiotics are long proposed to have positive health effects achieved through alterations in both composition and function of the host large bowel microbiome, and there is a large literature investigating obesity-related metabolic health outcomes (118). Mechanisms proposed include improved intestinal epithelial integrity decreased low grade endotoxemia-induced inflammatory response, and promotion of colonic short chain fatty acids (SCFA) including butyrate as a nutrient source for the large bowel colonocytes (119, 120). It has been reported both that probiotic effects of yogurt may modulate glycaemia and related endpoints, and exert beneficial effects (117), or conversely that the evidence is lacking. Astrup evaluated both cohort studies and RCTs, concluding that observational data supported the significant association between yogurt, decreased risk of body weight gain, and CVD, but that notably this evidence was supported only in part by RCTs. A later 2017 review that reported on 7 RCTs, in addition to animal model evidence, in turn also concluded that the effects of probiotics on glycemic control were conflicting (121). A 2016 narrative review (122) of probiotics, prebiotics and combination symbiotic RCTs concluded that probiotics have only a small (~3%) effect on metabolic endpoints, with outcomes maximized when consumed within fermented milks or yogurts over an extended period of at least 8 weeks.

An important question is whether there is an independent causative relationship between probiotics *per se* and improvements in CVD, T2D or other metabolic health outcomes, independent of the food matrix such as yogurt. Notably, a recent systematic review and meta-analysis of RCTs evaluated the effects of probiotic yogurt vs. control yogurt on glycemic outcomes in obese individuals and those with T2D and reported no additional benefits of the probiotic yogurt (123). A comprehensive recent systematic review and meta-analysis of 20 prospective cohort studies and 52 RCTs (124) has highlighted the positive effects of fermented milks, yogurts and probiotics. The review reported fermented milk consumption to be associated with decreased CV risk, with probiotics added into dairy matrices lowering serum lipid profile, while yogurt consumption was associated with decreased T2D and metabolic syndrome risk. The authors did note however that heterogeneity of these multiple studies including variability in probiotic strains requires the outcomes to be interpreted with caution.

#### Homogenisation

Cow's milk is commonly homogenized in order to increase the physical stability of the food product. It aids in the prevention of separation of water and lipid components into bilayers through a decrease in lipid droplet size and incorporation of dairy protein into the droplet interface. The decrease in milk fat globule size, essentially a disruption in the MFGM, which allows them to be dispersed uniformly through the milk product, has been proposed to result in improved digestibility and potentially health outcomes (125). The recent review by Mozaffarian et al. (22) noted that homogenization may destroy MFGM, and proposed that this may have implications for the cardiometabolic effects of dairy lipids. They cited an 8 weeks RCT which compared whipping cream (intact MFGM) with energy-, total dairy- and SFA-matched butter fat (homogenized, decreased MFGM) (67). Butter fat worsened both LDL-C and apolipoprotein B:A-I ratio, with no effects during whipping cream intervention.

## Dairy and the Saturated Fat Controversy

The high saturated fatty acid (SFA) content of dairy and its myriad of food products has long raised concern (126), aligned with international guidelines (126, 127) to minimize dietary intake of animal-origin SFAs to achieve better cardiometabolic health. Early systematic reviews supported a relationship between dietary fatty acids and serum lipids, and also serum lipids and CV morbidity and mortality, as recently summarized by Hooper et al. (128). Mechanisms by which animal-origin SFAs may adversely affect circulating lipids include inhibiting LDL-receptor removal of lipoproteins from circulation, enhancing secretion of ApoB100-containing lipoproteins VLDL and LDL into circulating, and packing excess cholesterol in LDL particles; hence in combination increasing circulating LDL-C, TC and LDL-C:HDL-C ratio concentrations. In their Cochrane systematic review these authors in turn have shown a prolonged (2 years+) decrease in dietary SFA to be associated with a significant decrease in combined CV events, with some evidence of a dose-relationship (128). Replacing SFA with PUFA, but possibly not MUFA, and/or carbohydrate has been proposed as the most successful approach. However, over the past 10 years a consensus of studies has built which has questioned the assumption of adverse effects for dairy-origin SFAs for both CV and T2D outcomes. Notably, it is no longer adequate to consider nutrients in isolation, with evidence that the complex matrix of a food may be equally or more important than the fatty acid content and composition alone when predicting cardiometabolic risk (3, 10, 11). It has been proposed that in a complex dairy food such as cheese, for example, the effect of SFAs on blood lipids and disease risk may be counterbalanced by the content of protein, calcium, or other dietary components (23, 129). Aside growing evidence that dietary SFA-induced increases in LDL-cholesterol may not *per se* adversely affect cardiometabolic health (3).

Evidence that underpins the relationship between dairy fat and cardiometabolic outcomes to date exists predominantly from observational studies. This must be noted since association studies cannot determine cause and effect relationships, and are subject to confounding that may bias relationships between diet and disease outcomes. Human clinical trials investigating dairy interventions characteristically have been of short- or moderate- duration, with CV or T2D intermediary risk endpoints, and not disease incidence, evaluated. A summary of both is presented below.

### Observational Studies

***CVD:*** Contrary to prior concerns for high SFA animal-origin food groups (1), there is no consistent evidence from epidemiologic studies that a higher intake of dairy products is associated with increased CVD risk or incident CVD (3, 4, 6, 7, 20, 130). Key systematic reviews, meta-analyses, umbrella and narrative reviews are presented in Table 1a. In 2014 Astrup summarized observational studies which reported that the consumption of milk or dairy products was in fact inversely related to incidence of CVD (117), an outcome supported in a recent state of the art review (3). Inclusion of dairy products in the diet may ameliorate characteristics of adverse metabolic health including dyslipidemia, insulin resistance, hypertension, abdominal obesity, and the metabolic syndrome cluster (137), which together markedly increase the risk of both T2D and CVD. This had been proposed previously in meta-analyses of prospective cohort studies that identified an inverse association between milk and other dairy components and CV endpoints (4, 5, 116), with Elwood et al. originally noting a mismatch between this evidence and perceptions of harm from consumption of dairy (4). In 2015 a meta-analysis of prospective cohort studies provided further evidence supporting the beneficial effect of dairy consumption on CVD. Low-fat dairy products and cheese may protect against stroke or CHD incidence (6), in particular yogurt (134). A 2016 meta-analysis of prospective cohort studies again showed that dairy consumption may be associated with reduced risks of CVD, albeit with the authors noting that additional data are needed to more comprehensively examine potential dose-response patterns (7). It is now apparent that most meta-analyses report no association or even a weak inverse association between intake of dairy products and CVD endpoints (8, 20, 131, 133, 135, 136), or risk biomarkers including serum lipoprotein LDL-cholesterol. Characterized as convincing probable evidence, a recent 2020 umbrella review by Godos et al., reported a decreased association between total dairy and CVD (9). Arguably there is more controversy with respect to butter fat studies (2, 132). The recent large multinational Prospective Urban Rural Epidemiology (PURE) study from 21 countries in five continents has since also reported similar findings, with dairy intake associated with lower risk of mortality and major CVD events (130). However, even in light of the absence of a positive adverse association between dairy and CVD, there is some evidence that replacing dairy fat with PUFAs, especially from plant-origin foods, may be advantageous and confer positive health benefits (20).

**Table 1a:** Summary of meta analyses, systematic reviews and narrative reviews reporting CVD outcomes: observational studies.

**References**	**No. of studies**	**Study design**	**Dairy formats**	**Outcomes**
Elwood et al. (4)	Eleven studies prospective cohort	Meta-analysis	Total dairy, milk, butter, cheese	Inverse association for total dairy vs. IHD RR 0.92 (0.80, 0.99) and stroke RR 0.79 (0.68, 0.91). Mismatch between evidence from long-term prospective studies and perception of harm from consumption of dairy
Soedamah-Muthu et al. (5)	Seventeen studies Prospective cohort	Meta-analysis	Total dairy, low-fat, high-fat dairy	Modest inverse association for milk and CVD (4 studies) RR 0.94 per 200 mL/d, 95% CI: 0.89, 0.99; but not CHD (6 studies) RR 1.00, 95% CI: 0.96, 1.04; or stroke (6 studies) RR 0.87, 95% CI: 0.72, 1.05. No association for high-fat or low-fat dairy, per 200 g/d, with CHD.
Huth and Park (116)	Twenty-three studies Prospective cohort	Narrative review	Total dairy, low-fat, high-fat, milk, cheese, butter, yogurt	Most, but not all, showed no relationship or inverse association between dairy and risk of CVD and stroke
Astrup et al. (117)	Seventeen studies Prospective cohort	Narrative review	Milk, yogurt, mixed dairy	Modest inverse association between milk and risk of CVD, RR reduction 6%. Milk not associated with reduction in risk of CAD, stroke, or total mortality.
Qin et al. (6)	Twenty-two studies Prospective cohort	Meta-analysis	Total dairy, milk, low-fat dairy, high-fat dairy, cheese, butter, yogurt	Inverse association for total dairy and CVD (nine studies; RR 0.88, 95% CI: 0.81, 0.96), stroke (12 studies; RR 0.87, 95% CI: 0.77, 0.99), but not CHD
Alexander et al. (7)	Thirty-one studies Prospective cohort	Systematic review and meta-analysis	Total dairy, dairy products, low-fat dairy, high-fat dairy, Ca from dairy	Inverse association for total dairy (RR 0.91; 95 % CI 0.83, 0.99) and calcium from total dairy (RR = 0.69; 95 % CI 0.60, 0.81) and stroke; also cheese and CHD (RR 0.82; 95 % CI 0.72, 0.93) and stroke (RR 0.87; 95 % CI 0.77, 0.99). Low level evidence after adjustment for within-study covariance
Drouin-Chartier et al. (131)	Twenty-one studies Prospective cohort	Systematic review and meta-analysis	Total dairy, milk, high-fat dairy, low-fat dairy, cheese, yogurt, fermented	Favorable or neutral associations with CVD-related clinical outcomes. Moderate-quality evidence for total dairy as neutral for CVD risk High-quality evidence for total dairy and decreased hypertension risk
Pimpin et al. (132)	Fifteen studies Prospective cohort	Systematic review and meta-analysis	Butter fat	Not significantly associated with CVD
Guo et al. (133)	Twenty-nine studies Prospective cohort	Meta-analysis	Total dairy, low-fat dairy, high-fat dairy, milk	No associations for total (high-fat/low-fat) dairy, and milk with CHD or CVD
Wu and Sun (134)	Nine studies Prospective cohort	Meta-analysis	Yogurt	Highest category consumption ns related to incident CVD; RR 1.01, 95% CI 0.95, 1.08
Gholami et al. (8)	Twenty-seven studies Prospective cohort	Meta-analysis	Total dairy	Inverse association between total dairy and CVD; no relationship for CHD
Dehghan et al. (130)	PURE study	Prospective cohort	Whole-fat dairy: milk, yogurt, cheese	Inverse association between higher dairy and CV events HR:0.81; 95% CI:0.77, 0.93
Yu and Hu (20)	Four studies Prospective cohort	Narrative review, summarizing meta analyses of cohort studies for CVD endpoints	Total dairy, milk, cheese, yogurt	Null or weak inverse association between consumption of dairy products and risk of CVD. Milk RR 1.01, cheese RR 0.92, yogurt RR 1.03. Odd chain fats as biomarkers: inverse association of C15:0 and C17:0 with CVD
Guillocheau et al. (81)	Thirteen studies Prospective cohort	Narrative review of cross-sectional, case control, cohort studies	Monounsaturated *trans*-palmitoleic acid	Neutral or inverse associations; but inconsistent findings for CVD risk factors
Hirahatake and Astrup (135)	Seventeen studies Prospective cohort	Narrative review summarizing meta-analyses, systematic reviews, prospective cohort studies	Multiple dairy formats including full fat, low-fat, cheese, yogurt	Neutral or inverse association of full-fat dairy with CVD
Kim et al. (136)	Sixty-two studies Korean cohorts; 42 cross-sectional, 3 case-control, 17 cohort	Systematic review and meta-analysis	Total dairy, milk	Inverse association with CVD risk factors
Godos et al. (9)	Fifty-three studies Prospective cohort	Umbrella review of meta analyses	Total dairy, milk, high-fat dairy, low-fat dairy, cheese, butter, yogurt	Inverse association for total dairy and CVD; and for cheese and CHD, CVD and stroke risk factors in highest vs. lowest category; no association with milk

*CAD, coronary artery disease; CHD, coronary heart disease; CI, confidence interval; CVD, cardiovascular disease; HR, hazard ratio; IHD, ischemic heart disease; RR, relative risk*.

#### T2D

Whilst prospective cohort studies have commonly shown a range of animal products, for example red and processed meats, to be associated with increased risk of T2D (138), conversely numerous meta-analyses and systematic reviews report dairy food consumption to be linked to neutral or even decreased T2D risk (see Table 1b). These reviews utilize data from adult populations globally, reporting greatest effects with yogurt and cheese (19), in turn representative of both low-fat and high-fat dairy, respectively. The early meta-analysis of observational cohort studies showed higher total dairy also to be associated with a reduction in T2D risk (4), as did another small meta-analysis of 7 cohort studies (12). In 2013 Aune et al. published a meta-analysis of 17 prospective cohort studies, reporting that a higher consumption of total dairy products and cheese, as well as low-fat dairy, was associated with a lower risk for T2D (13). Further, investigation of dose-response showed a 7% decrease in T2D risk for every 400 g total dairy food consumed per day, which was estimated to be equivalent to ~1.7 servings of liquid milk per day (13). A second meta-analysis of 14 prospective cohort studies confirmed a similar 6% decrease in T2D risk for every 200 g of total dairy per day, plus in turn a 12% decrease for every 200 g low-fat dairy per day (14). This increased further to a 20% lower risk in T2D for every 30 g of cheese, and a 9% lower risk associated with consuming 50 g per day of fermented yogurt showing an inverse association between dairy intake and risk of T2D. Gijsbers et al., meta-analysis also reported an inverse association between dairy intake and risk of T2D (16), as did Schwingshackl et al. (139) and Soedamah-Muthu et al. (140). An updated recent 2019 meta-analysis of 12 studies has also reported total dairy consumption to be associated with lower risk of T2D, with moderate effects of cheese and strong effects of yogurt and low-fat dairy (17), in supported by a 2020 review of 12 prospective cohort studies (9). Notably however, in a combined analysis of men and women from the US Health Professionals Follow-Up Study and the US Nurses' Health Study I and II, no relationships were observed with total dairy, low-fat dairy or high-fat dairy, with only yogurt intake associated with a decreased risk of T2D (15). Also contrary to earlier findings of a significant association with total dairy in these large US cohorts. The authors proposed the longer follow up (+10 years) may account for this change in outcome, in turn observed in a meta-analysis of prior cohort studies published by their research team where benefits of dairy intake were diminished in long follow up studies (15). Pimpin et al. reported an inverse association with incidence of T2D in their more recent meta-analysis of butter fat (132).

**Table 1b:** Summary of meta analyses, systematic reviews and narrative reviews reporting T2D outcomes: observational studies.

**References**	**No of studies**	**Study design**	**Dairy formats**	**Outcomes**
Elwood et al. (4)	Five studies Prospective cohort	Meta-analysis	Total dairy, milk	T2D incidence 3.9%. Inverse association for total dairy and incident T2D in highest relative to lowest intake (RR 0.85; 95% CI 0.75, 0.96)
Tong et al. (12)	Seven studies Cohort	Meta-analysis	Total dairy, milk, high-fat dairy, low-fat dairy, yogurt	T2D incidence 4.0%. Inverse association for total dairy (RR 0.86; 95% CI 0.79, 0.92), low-fat dairy (RR 0.82; 0.74, 0.90), whole milk (RR 0.95; 0.86, 1.05), yogurt (RR 0.83; 0.74–0.93) and T2D; neutral for high-fat dairy (RR 1.00; 0.89, 1.10) and T2D
Aune et al. (13)	Seventeen studies Prospective cohort	Systematic review and meta-analysis	Total dairy, milk, high-fat dairy, low-fat dairy, yogurt	T2D incidence 5.7%. Inverse association for total dairy (400 g/d; RR 0.93; 95% CI 0.87, 0.99), high-fat dairy (200 g/d; RR 0.98; 0.94, 1.03), low-fat dairy (200 g/d; RR 0.91; 0.86, 0.96), milk (200 g/d; RR 0.87; 0.72, 1.04), cheese (50 g/d; RR 0.92; 0.86, 0.99), yogurt (200 g/d, RR 0.78; 0.60, 1.02) and T2D
Gao et al. (14)	Fourteen studies Prospective cohort and case control	Systematic review and meta-analysis	Total dairy, milk, high-fat dairy, low-fat dairy, yogurt	T2D incidence 5.6%. Inverse association for total dairy (200 g/d; RR 0.94; 95% CI 0.91, 0.97), low-fat dairy (200 g/d; RR 0.88; 0.84, 0.93), cheese (30 g/d; RR 0.80; 0.69, 0.93), yogurt (50 g/d; RR 0.91; 0.82, 1.00) and T2D
Chen et al. (15)	Fourteen studies Prospective cohort	Meta-analysis	Total dairy, milk, high-fat dairy, low-fat dairy, yogurt	T2D incidence 7.8%. Neutral association for total dairy (1 serve/d; RR 0.98; 95% CI 0.96, 1.01), inverse association for yogurt (1 serve/d; RR 0.82; 0.70, 0.96) and T2D
Gijsbers et al. (16)	Twenty-two studies Prospective cohort	Meta-analysis	Total dairy, milk, high-fat dairy, low-fat dairy, yogurt	T2D incidence 7.4%. Neutral or inverse association for total dairy (per 200 g/d, RR 0.97; 95% CI 0.95, 1.00), low-fat dairy (per 200 g/d; RR 0.96; 0.92, 1.0), yogurt (per 80 g/d; RR 0.86; 0.83, 0.90), ice cream (per 10 g/d; RR 0.81; 0.78, 0.85) and T2D
Pimpin et al. (132)	Eleven studies Prospective cohort	Meta-analysis	Butter	T2D incidence 3.8%. Inverse association for butter (RR 0.96; 95% CI 0.93, 0.99) and T2D
Drouin-Chartier et al. (131)	Seven studies Prospective cohort	Systematic review of meta analyses	Total dairy, high-fat dairy, low-fat dairy, dairy fat, milk, cheese, yogurt, fermented	High-quality evidence for inverse association of low-fat dairy and yogurt; moderate-quality evidence for total dairy and cheese; high to moderate evidence for high-fat dairy, milk, fermented dairy and T2D
Schwingshackl et al. (139)	Twenty-one studies Prospective cohort	Systematic review and meta-analysis	Dairy as food group; total dairy, high-fat dairy, low-fat dairy	T2D incidence 7.8%. Inverse association with total dairy (RR 0.91; 95% CI 0.85, 0.97) in highest vs. lowest category, but only in Asian and Australian not American and European studies; borderline inverse association for low-fat dairy, no association for high-fat dairy and T2D
Riserus and Marklund (63)	Nine studies Prospective cohort	Narrative review	Circulating dairy odd chain dairy fatty acids (OCFA), C15:0, C17:0	Inverse association of OCFA biomarkers with T2D, but not CVD; may reflect high-fat dairy only
Soedamah-Muthu and de Goede (140)	Twenty-six studies Prospective cohort	Systematic review and meta-analysis	Total dairy, low-fat dairy, yogurt	T2D incidence 0.8%. Inverse association total dairy (RR 0.97; 95% CI 0.95, 1.00), low-fat dairy (RR 0.96; 0.92, 1.00), yogurt (80 g/d RR 0.86; 0.83, 0.90).
Alvarez-Bueno et al. (17)	Twelve studies Cohort and case control	Systematic review of meta analyses	Total dairy, low-fat dairy, milk, yogurt	T2D incidence 7.4–7.8%. Inverse association for total dairy (RR range 0.86–0.91), low-fat dairy (RR range 0.80–0.83), low-fat milk (RR 0.82), yogurt (RR range 0.74–0.86), and T2D
Godos et al. (9)	Fifty-three studies Prospective cohort	Umbrella review of meta analyses	Total dairy, milk, high-fat dairy, low-0 fat dairy, cheese, butter, yogurt	“Possible” (evidence level) inverse association of total dairy, cheese, butter, yogurt; no significant association for milk and T2D
Imamura et al. (58)	Sixteen studies Prospective cohort	Meta-analysis	Circulating or adipose tissue dairy odd-chain fatty acids (OCFA) C15:0, C17:0, *trans*-palmitoleic acid	T2D incidence 23.8%. Inverse association for C15:0 HR 0.80; 95% CI, 0.73, 0.87; C17:0 HR 0.65; 0.59, 0.72; *trans*16:1-n7 HR 0.82; 0.70, 0.96; sum OCFA HR 0.71; 0.63, 0.79, and T2D

*CI, confidence interval; HR, hazard ratio; IHD, ischemic heart disease; OCFA, odd chain fatty acids; RR, relative risk; T2D, type 2 diabetes*.

Other individual large prospective cohort studies in the US support a neutral, not adverse, relationship between total dairy and risk of T2D including postmenopausal women from the Women's Health Initiative Observational Study. Whilst higher dairy food consumption was associated with 40–50% reduced risk, it was notable that these large effects were related to low-fat dairy and yogurt intake (141). Prospective cohort studies conducted outside the US have also found beneficial or neutral effects of total dairy food consumption, as well as for specific dairy food groups which include high-fat dairy and cheese in addition to low-fat dairy or yogurt, on T2D risk in Britain, Europe, Japan and Australia (142–149). This was confirmed in a recent expert panel position paper (18), however, it remains important to note that the possibility of confounding is an aspect of observational studies which requires their review alongside well-designed and conducted RCTs in order to draw balanced consensus.

Overall, there is an extensive body of data from large observational trials of long duration, gathered over the last 20+ years, that support the association between total dairy intake and decreased incidence of CVD and/or T2D. Meta-analysis of prospective cohort studies of CVD-related incidence supports the recent characterization of this evidence as “convincing probable.” Whilst it is not as yet clear as to dose-response patterns for dairy and CVD, there are strong associations that suggest different relationships with different dairy formats, specifically liquid milk, butter, cheese and yogurt. Similar observations are reported for T2D incidence. As noted above, confounding remains of concern if evidence is obtained solely from observational data. There is far less data available from human interventions trials from which consensus might be built on the relationships of CVD or T2D with total dairy or the dairy food groups milk, cheese, butter and yogurt. In particular there is an absence of dairy RCTs designed to evaluate endpoint CVD and T2D events, required in order to conclude that totality of evidence is convincing.

### Randomized Controlled Trials (RCTs)

#### CVD

There is far less data available from RCTs from which consensus might be built on the relationships of CVD with total dairy or the dairy food groups milk, cheese, butter and yogurt (see Table 1c). Huth and Park (116) originally reported on short-term intervention studies and lipid biomarkers, where diets higher in SFA from whole milk and butter adversely increased LDL-C when substituted for carbohydrate or USFA. Adverse effects of high SFA vs high USFA (PUFA+MUFA) butter fat on lipid parameters of lean healthy men have previously been reported from our laboratory (150, 151). Meta-analysis (116) however also showed increased cardio-protective HDL-C, resulting in little or no worsening of the key CVD marker total cholesterol:HDL-C ratio. In addition cheese, when matched to the total and SFA content of butter, was shown to result in significant LDL-C lowering (116), confirming the outcomes from prior observational data. Other authors have since also questioned the role of dairy SFAs in CVD, with evidence that substitution of dairy SFA with (i) plant-derived n-6 PUFA vegetable oils (unless balanced with n-3 PUFA), and (ii) carbohydrate with high glycemic index (GI), may also worsen adverse cardiometabolic endpoints (117). Drouin-Chartier et al. more recently conducted an extensive umbrella review of data obtained from meta-analyses of RCTs, in addition to a large number individual RCTs. Whilst noting that these trials were conducted predominantly in healthy individuals, contrary to Huth and Park (116) they reported no adverse impact of high-SFA dairy consumption on multiple cardiometabolic variables, including various blood lipid fractions, blood pressure, inflammation and vascular function (126). The authors noted that their review narrative failed to address several potential confounders, including change in body weight, the multiple and varied control arms (both dairy and non-dairy dietary components) used in these dairy interventions, and the possibility of inadequate power in multiple trials where the CVD outcomes assessed were not primary end points. Nor did they evaluate potential publication bias. However, the conclusions drawn from this review, that there was no evidence from RCTs to support a detrimental effect of regular- or high-fat dairy on these many CVD risk factors, was in agreement with the observational trials discussed above.

**Table 1c:** Summary of meta analyses, systematic reviews and narrative reviews reporting CVD outcomes: RCTs.

**References**	**No of studies**	**Study design**	**Dairy formats**	**Outcomes**
Huth and Park (116)		Narrative review of RCTs and meta analyses	Total dairy, low-fat, high-fat, milk, cheese, butter, yogurt	Milk and butter increased plasma lipids (TC, LDL-C, HDL-C, apo B) in short term RCTs; however cheese, matched to butter for total-fat and SFA, significantly or borderline decreased LDL-C
Brouwer et al. (75)	39 studies: 29 industrial *trans* + 17 CLA + 6 ruminant fat RCTs	Meta analysis	*Trans*-fats vs. isoenergetic *cis-*monounsaturated fatty acids (MUFA, calculated)	Industrial *trans* fatty acids increased LDL-C:HDL-C ratio by 0.055 (95% CI 0.044, 0.066) vs. *cis* MUFA; by 0.038 (0.012, 0.065) vs. ruminant *trans* fatty acids, and 0.043 (0.012, 0.074) vs. CLA, for each energy % replaced
Brouwer et al. (80)	48 CLA + 11 ruminant fat RCTs	Meta-analysis	*Trans*-fats vs. *cis*-control fats	Ruminant *trans* fat and CLA both significantly increased LDL-C, LDL:HDL ratio, TC:HDL ratio vs. *cis*-control fats; CLA also decreased HDL-C vs. *cis*-control fats
Astrup et al. (117)		Narrative review	Yogurt, dairy	Yogurt and other dairy products decreased risk of CVD; supportive evidence but not conclusive.
Derakhshande-Rishehri et al. (95)	33 studies: 23 supplement + 10 food enriched RCTs	Systematic review and meta-analysis	CLA	Foods enriched with CLA or CLA supplements decreased LDL-C
Drouin Chartier, et al. (126)		Narrative review; umbrella review of meta analyses	Total dairy, high-fat, low-fat, milk, yogurt, cheese	Total or high-fat dairy, neutral effect (not adverse) on CVD lipid risk factors. Adverse effects of SFAs attenuated in complex food matrices of milk, cheese, yogurt
Wirunsawanya et al. (30)	9 RCTs	Systematic review and meta-analysis	Whey protein	Whey improved CVD risk factors in overweight and obese, but confounded by body weight and fat mass loss
Haghighatoost et al. (96)	11 RCTs	Systematic review and meta-analysis	CLA	CLA supplementation may worsen acute phase protein CRP and pro-inflammatory cytokine TNF-α
Li et al. (89)	3 RCTs	Narrative review	Uncommon dairy-origin fatty acids, including furan fatty acids, n-3 docosapentaenoic acid (DPA), and conjugated fatty acids	DPA has favorable effect on CV endpoints. Furan fatty acids and CLNA may be beneficial for CV health, but clinical evidence limited. CLA variable outcomes in risk factors RCTs, but no trials of CVD incidence.
Badeley et al. (32)	37 RCTs	Systematic review and meta-analysis	Whey protein	Whey significantly decreased SBP, DBP, waist circumference, HDL-C and TG; confounded by central fat mass (waist) loss

*Apo B, apolipoprotein B; CI, confidence interval; CLA, conjugated linoleic acid; CLNA, conjugated linolenic acid; CRP, C-reactive protein; CVD, cardiovascular disease; DBP, diastolic blood pressure; DPA, docosapentaenoic acid; HR, hazard ratio; IHD, ischemic heart disease; HDL-C, high density lipoprotein –cholesterol; LDL-C, low density lipoprotein –cholesterol; MUFA, monounsaturated fatty acid; OCFA, odd chain fatty acids; RCT, randomized controlled trial; RR, relative risk; SBP, systolic blood pressure; SFA, saturated fatty acid; TC, total cholesterol; TG, triglyceride; TNF-α, tumor necrosis factor-α; T2D, type 2 diabetes*.

Table 1c also summarizes meta-analyses of RCTs investigating effects of various specific dairy components on CVD risk factors. Brouwer et al. (75, 80) have reported adverse effects of *trans*-fats in large meta-analyses of up to 48 industrial CLA and 11 ruminant *trans*-fat RCTs. Various authors have reported adverse (96), positive (87), and mixed (89) effects of CLA on CV endpoints. Whilst positive effects have been reported in several meta-analyses of whey protein RCTs (30, 32).

A major gap in the interpretation of this evidence is the absence of large, long-term dairy RCTs where CVD incidence is the primary outcome. To date, the majority of data reported has been from short-term interventions in healthy individuals rather than those as high risk, and has assessed accessible endpoints of CV risk. Only very few long-term interventions, such as the Finnish Dietary Prevention of Coronary Heart Disease studies for men and for women (152, 153), cluster-randomized 12 years intervention trials targeting decreased SFA through dairy replacement, have investigated CVD endpoints. These was conducted in two long-stay hospital wards where the majority of dairy fats were replaced by plant-origin n-6 PUFA fats for a 6 years duration, prior to crossover. Notably there was a significant decrease in serum cholesterol and incident CHD in men, smaller in women, when dairy was largely replaced by PUFA. The conundrum as to whether this decrease in clinical events was driven by decreasing SFA (dairy products replaced included whole milk and butter) or increasing PUFA (dairy substitutes of soybean oil in skimmed milk and high PUFA “soft” margarine) cannot be untangled. The critical nature of the comparator (or “control”) arm in the interpretation of such trials has recently been highlighted in the wider discussion concerning the role of dietary SFA in prevention of CVD (154). In a systematic review of large dietary interventions, analyzing the data sets on the basis of SFA being replaced by PUFA, or CHO or by ignoring the composition of the substitute, led to quite different positive (PUFA) or null (CHO, ignore) relationships between SFA and CVD. This clearly highlights the need to conduct long duration RCTs trials where the non-dairy control arm is carefully planned and evaluated.

#### T2D

Again, as noted previously there is little data available from long-term RCTs that directly attribute amelioration of T2D risk factors, or prevention of T2D, through dairy intake. Rideout et al. conducted a small sample size, cross-over study of 2 x 6 months duration which reported improved insulin sensitivity (155), although notably this was low-fat dairy. A second cross-over trial also of 2 × 6 months duration conversely reported no effect of a high vs. low dairy intake on glycemic endpoints (156). A more recent study in hyperinsulinaemic adults that compared 6 weeks supplementation of adequate (three servings/week) vs. high (three servings/week) dairy intake however did not observe any difference in glucose or insulin outcomes including insulin sensitivity, insulin secretion, and β-cell function (157). Table 1d presents a summary of systematic reviews, meta-analyses and narrative reviews. A systematic review of 10 RCTs of 1 week to 6 months duration, notably in weight stable individuals, failed to find conclusive evidence of improvement or detriment of dairy consumption on glycemic endpoints (158). Only 4 of 10 RCTs had a positive outcome on insulin sensitivity assessed as HOMA-IR. Pasin and Commerford reported on a meta-analysis of 28 dairy RCTs, with limited evidence of fermented dairy, probiotics and dairy proteins improving glycemic control (27). A more recent narrative review of 15 RCTs, of 2 weeks to 6 months duration, in individuals at risk of T2D concluded that whilst dairy does not have detrimental effects on glucose-related outcomes, positive outcomes are not universal (19). High dairy consumption was compared to a variety of no dairy and low dairy treatments. 10 of the studies showed no improvements with high dairy intake, whilst 5 studies showed improved HbA_1c_, plasma glucose, and insulin resistance (HOMA-IR). O'Connor et al. have also recently published a review of 38 RCTs, in a heterogeneous meta-analysis of children (>5 years) and adults without T2D, where glycemic endpoints were measured in trials comprising a higher vs. lower dairy arm comparison (159). Notably elevated dairy intake was associated with worsening FPG, but not fasting insulin or HbA_1c_, with the authors recording that the quality of evidence was low with high uncertainty. Sohol et al., have also recently conducted a meta-analysis of 30 RCTs (160) which reported that dairy, in particular low-fat dairy, decreased HOMA-IR assessed insulin resistance. The authors noted a series of limitations of their analysis including absence of discrimination between dairy food groups of milk, cheese, yogurt and components including dairy proteins, Ca and vitamin D, as well as missing data for HOMA-IR analyses in the original papers and absence of baseline glycemic status. They also noted the significant heterogeneity in the RCTs included in the meta-analysis. Table 1d also summarizes a number of other reviews of RCTs where effects of the individual dairy components CLA and whey protein have been assessed. RCTs investigating effects of CLA (88, 97) have reported no significant positive effects on glycemic endpoints, whilst whey protein interventions (30, 32) have caused a significant decreased in FPG.

**Table 1d:** Summary of meta analyses, systematic reviews and narrative reviews reporting T2D outcomes: RCTs.

**References**	**No of studies**	**Study design**	**Dairy formats**	**Outcomes**
Turner et al. (158)	10 RCTs healthy and T2D risk, metabolic syndrome	Systematic review	Total dairy; no other lifestyle or other dietary change, including no weight change	4 studies had positive effect, 1 negative effect, and 5 no effect on homeostasis model assessment of insulin sensitivity (HOMA-IS)
Pasin and Comerford (27)	28 RCTs: 12 dairy foods + 16 dairy proteins; T2D and non-T2D	Meta-analysis	Milk, cheese, yogurt, whey protein, casein	Whey and casein improved glycemic endpoints (limited evidence ≤50 g/d protein). Cultured (yogurt and fermented drink “dough”) and non-cultured (milk, cottage cheese) improved glycemic status in T2D; addition of probiotics further improved glycaemia, but limited evidence
Rahbar et al. (88)	32 RCTs healthy	Meta-analysis	CLA (*cis*9, *trans*11; *trans*10, *cis*12) supplements and enriched foods	CLA supplements (standardized mean difference, SMD, 0.075 mg/dL; 95% CI −0.099, 0.249) or CLA-enriched foods (SMD 0.126 mg/dL; 95% CI −0.100, 0.352) had no significant effect on FBG
Wirunsawanya et al. (30)	9 RCTs: 4 with glucose endpoints T2D risk ow/obese	Meta-analysis	Whey protein	Whey significantly decreased FBG; pooled mean difference (MD) 0.76; 95% CI 0.14, 1.38)
Mitri et al. (19)	15 RCTs T2D risk, ow/obese or metabolic syndrome	Narrative review	Total dairy vs. limited or no dairy; 8 studies weight maintenance	10 studies neutral effect, 5 studies improved glycemic endpoints including HbA_1c_, FPG, insulin, HOMA-IR.
O'Connor et al. (159)	34 RCTs non-T2D	Systematic review and meta-analysis	Ruminant-origin dairy products elevated vs. low intake	Elevated dairy intake positively associated with FPG, [mean difference MD 0.07 mmol/L; 95% CI 0.01, 0.12], but not fasting insulin (MD −2.97 pmol/L; −7.05, 1.10) or HOMA-IR (SMD −0.07; −0.26, 0.12); negatively associated with HbA_1c_ in 4 studies (MD −0.09%; −0.09, −0.03). Quality of evidence low, high uncertainty
Sochol et al. (160)	30 RCTs	Systematic review and meta-analysis	Total dairy, milk, butter, cheese, yogurt	Diet with low-fat dairy decreases insulin resistance (HOMA-IR); mean difference (MD) −1.21 (95% CI−1.74, −0.67); may be confounded by decreased body weight and waist circumference
Badeley et al. (32)	37 RCTS ow/obese	Systematic review and meta-analysis	Whey protein isolate, concentrate, extract, supplement, powder, hydrolysate	Whey decreased FBS; weighted mean difference (WMD)−1.42, 95% CI 1.52, −1.31
den Hartigh (97)	13 RCTs, 5 with glucose endpoints, healthy ow/obese	Narrative review	CLA (*cis*9, *trans*11; *trans*10, *cis*12) supplements and dairy	CLA decreased body weight and body fat, but no glycemic improvement. May improve insulin resistance in high risk adults/children. In abdominally obese men, adverse increase in fasting glucose, insulin and CRP, and decreased insulin sensitivity.

*CI, confidence interval; CLA, conjugated linoleic acid; FBG, fasting blood glucose; HOMA-IR, homeostatic model assessment-insulin resistance; HOMA-IS, homeostatic model assessment-insulin sensitivity; Mean difference, MD; RCT, randomized controlled trial; o/w, overweight; T2D, type 2 diabetes; SMD, standardized mean difference; WMD, weighted mean difference*.

Again, a major omission in the interpretation of these RCTs is the fact that large, long-term interventions investigating effect of dairy on T2D incidence have not been conducted. To date, the majority of data reported has been from short-term interventions, of both healthy and impaired glucose tolerant individuals assessing commonly measured glycemic endpoints.

## Protective Effects of Dairy Food Matrices

### Yogurt, Cheese

As referred to in the sections above, the complex matrix of dairy has in recent years been proposed to significantly modulate the nutritional properties of these food products (10, 25, 26, 161), acting both on acute and chronic endpoints. In 2017, a primarily Europe-based, expert panel concluded that evidence from RCTs supported the proposal that intake of whole dairy foods vs. single dairy constituents resulted in different cardiometabolic effects (25), and that this may underpin the protective inverse relationships commonly reported for fermented dairy products, yogurt and cheese. They proposed that food structures and processing methods may enhance interactions between nutrients in the dairy matrix, such that the nutritional value of the product could not be directly related to the simple nutrient content, and introducing the concept of “biofunctionality” of nutrients within dairy food structures. An example of a recent intervention study in healthy adults, where high-fat meals with matched total fat content were delivered across a range of dairy product formats, and where quite different postprandial response of serum lipid and lipoprotein fractions was observed (161).

Cheese provides a clear example, where the predicted adverse effects of SFAs on cardiometabolic health are ameliorated when these FAs are consumed as part of this complex high-fat matrix (10, 126, 129). These outcomes were unexpected in early studies where high-SFA dairy fat when given as cheese failed to raise total- and LDL-cholesterol compared with low-SFA control diets (162) or compared with butter fat (163). Evidence has grown in support of cheese being a neutral food group that fits into a healthy diet (164). This has recently been supported in an expert panel position paper where food matrix was reported to be a stronger determinant of health effects than SFA content *per se* (18). Cheese is manufactured in a wide range of formats which in turn themselves vary considerably in terms of food matrix, with corresponding variability in physiological response. For example, a recent acute study of commercial cheeses showed a soft format “cream” cheese to induce a greater increase in postprandial triglyceridaemia than butter, whilst hard format cheddar cheese did not. Purported to be linked to variance in GI disintegration processes (26). A longer-term RCT showed that dairy fat consumed within a cheese matrix lowered lipid markers vs. dairy fat delivered within alternative matrices (10).

In addition to cheese, there is evidence also that yogurt does not exert the adverse effects on blood lipids predicted solely by content of SFA (116, 117). In turn, the expert panel noted that consumption of yogurt is associated with decreased risk of T2D and cardiometabolic disease (18), as discussed previously in this review. Whilst a number of factors may contribute including low-total fat content, fermentation process and probiotic content, food matrix has been proposed as an additional significant contributor.

The mechanisms proposed to underpin these effects of food matrix are varied. Dairy products are highly varied themselves, with a vast array of both structure and processing methods used in production. Cheese is a good example. Based on macronutrient content cheese is a high-fat dairy product, as is butter, yet based on structural composition it is more similar to yogurt or liquid milk with a high protein, high calcium and high MFGM content (25). Decreased gastrointestinal (GI) absorption of lipid from dairy products rich in calcium, phosphorus and MFGM has been shown to directly increase lipid losses from the GI tract and so in turn decrease circulating lipid levels including chylomicron (diet-origin) TGs (165). Calcium within the dairy matrix has also been implicated in the suppression of LDL-cholesterol observed during chronic consumption of high fat cheese (25). Calcium phosphate has long been known to increase excretion of fecal bile acids, a mechanism by which LDL-cholesterol is suppressed since bile acids are synthesized from hepatic cholesterol and represent the primary pathway for cholesterol catabolism. It is less clear whether MFGM alone also affects fecal fat excretion, with mechanisms underpinning improvement in lipoprotein profile observed when, for example high-MFGM butter fat is consumed, being decreased GI cholesterol absorption and/or the regulation of expression of genes (25). Fermented dairy also has beneficial effects on blood lipids, hypothesized to be due to favorable effects on gut microbiota resulting in favorable SCFA profile. It has been confirmed that both yogurt and cheese increase levels of fecal SCFAs, with cheese intake increasing fecal butyrate, propionate, and malonate and decreasing fecal acetate and glycerol concentrations (25). Furthermore, there were significant correlations between fecal propionate and butyrate levels and LDL-cholesterol concentrations.

## Discussion

The contribution of dairy foods to cardiometabolic health has long been controversial. In particular, adverse effects that animal-origin dairy *trans* and SFAs may have on T2D and CVD outcomes. Consensus on the components of dairy in the diet has gradually been building, with some evidence of positive associations of whey protein and casein, milk fat globule membrane (MFGM) and polar phospholipids, vitamin D, and calcium with intermediary cardiometabolic endpoints. Conversely there is no consensus in support of the proposal that both *trans* and saturated dairy lipids have a consistent adverse effect on cardiometabolic health. Cohort studies report a neutral effect of *trans*-VA, but are not supported by RCT evidence that shows fatty acids containing *trans* double bonds to adversely alter serum cholesterol, irrespective of ruminant or industrial origin. In RCTs CLA both significantly improves and worsens CV risk factors but with little effect on glycaemia, and limited association with improved CV incidence reported in observational studies. For dairy SFA, evidence has been growing in support of no association or even a protective inverse relationship with cardiometabolic health, but largely driven by observational cohort studies. A clear research gap is the limited causal evidence of improvements in disease incidence obtained from intervention trials, with RCT evidence obtained predominantly from health individuals in trials of only short- or moderate-duration. In addition, it is proposed that the predicted adverse effects of these dairy lipids may be ameliorated when consumed within the complex food matrices of liquid milk, fermented cheese or yogurt.

In conducting this wide ranging review of the relationships between dairy and its myriad associated foods and components and cardiometabolic health, there are necessarily a number of limitations. Firstly this is a narrative and not systematic review, hence the ability to formally evaluate bias in its many forms in the large number of publications included both as individual studies or contributing to other narrative or systematic reviews is limited. The quality of the observational studies reviewed was highly variable, as were the design parameters of the RCTs. Study participants age, gender, anthropometric indices, health status and cardiometabolic risk factors also varied greatly between trials, both within and between within study type. It is not clear whether benefits are greatest in individuals identified as at risk of later disease. In RCTs sample size, composition of the comparator (“control”) arm, baseline dairy intake, dairy product and comparator dose, and duration of intervention are examples of important parameters that are not adjusted for in a narrative review.

Currently, there is little consensus by International dietary guidelines on the recommended fat content of dairy, and whether continued focus on consumption of low-fat dairy is warranted. The issue is complex, with both the quality and quantity of lipids contributed to the diet through dairy needing to be considered, with potential for higher fat products to promote a high energy density diet (166, 167) and body weight gain (168). Dietary diversity, i.e., “eat a variety of foods” has become a gradually accepted recommendation to promote a healthy diet and reduce risk of cardiometabolic disease, however recent evidence from cohort studies has shown this greater diversity to be associated with poor eating patterns and in turn weight gain and obesity. For example, a higher intake of processed foods, refined grains and also sugar sweetened beverages (SSBs), and a lower intake of unprocessed foods such as fish, fruits, and vegetables (169).

Table 2 presents the current dietary advice from both US and European medical associations for the prevention of CVD and T2D, where low-fat dairy is most commonly included. The American Heart Association (AHA) diet and lifestyle recommendations (170, 176), amongst others, continue to promote a healthy eating pattern that emphasizes low-fat dairy products alongside an adequate intake of plant foods including vegetable oils, nuts, whole grains, legumes, fish and poultry, and limited intake of sweets, SSBs and red meat. Conversely, the American Diabetes Association (ADA) annual guidelines continue to recommend a breadth of dairy products as part of a healthy diet, including milk and yogurt, for those with T2D (171), with no specific promotion of low-fat dairy. The European Society for Cardiology (ESC) guidelines (172) and the European Association for the Study of Diabetes (EASD) diabetes and nutrition study group (DNSG) (173) guidelines, expected to be updated in 2020 (177), both provide umbrella recommendations for SFAs to provide <10 en% and for low *trans*-fats. Alongside other healthy eating recommendations of consuming a diet high in vegetables, legumes, fruits and wholegrain cereals, EASD makes with no specific reference to dairy, whilst ESC recommends components of the Mediterranean Diet with minimal dairy for CVD prevention, and low-fat dairy for treatment of hypertension. A joint consensus report on management of hyperglycemia from ADA (174) and EASD (175) published concurrently in 2018 included diet and physical activity recommendations, with focus on medical nutrition therapy (MNT) of both energy restriction and optimal dietary quality including vegetables, legumes, fruits and whole grains, in addition to inclusion of dairy. This is part of a “no-one-size-fits-all” answer to diet and T2D by the ADA in their most recent 2019 Consensus Report (171).

**Table 2:** Current dietary advice from both US and European medical associations for the prevention of CVD and T2D.

**Medical Association**	**Year**	**Total fat**	**SFA**	**Dairy**	**Dietary advice**
AHA Eckel et al. (170)	2013		5-6%, decrease SFAs including from dairy. Decrease *trans* fats	Include low-fat dairy	Adequate intake of plant foods including vegetable oils, nuts, whole grains, legumes, fish, poultry, and limited intake of sweets, sugar sweetened beverages and red meat.
ADA American-Diabetes-Association (171)	2019	20–35 en%	Should be limited, replace with USFA. No *trans* fats.	Include dairy—milk and yogurt	SFA replaced with USFA, and not refined CHOs. CHO intake should emphasize nutrient-dense high fiber, including vegetables, fruits, legumes, whole grains, as well as dairy products. No “one-size-fits-all” and no single ideal ratio of CHOs, fats, and proteins for T2D; meal plans should be individualized.
ESC Piepoli et al. (172)	2016	Focus on fat quality, not total fat	<10 en%, replace with USFA	Include low-fat dairy	Low in SFA replaced with PUFA; with focus on wholegrain products, vegetables, fruit, nuts and fish.
EASD Mann et al. (173)	2004	<35 en%	<10 en%	No dairy recommendations	Range of macronutrient ratios, comprising 45–60 en% CHO, 10–20 en% protein; include vegetables, legumes, fruits, wholegrains.
ADA/EASD joint consensus report (174, 175)	2018	20–35 en%	Should be limited, replace with USFA. No *trans* fats.	Include dairy—milk and yogurt	SFA replaced with USFA, and not refined CHOs. Medical nutrition therapy (MNT) comprising energy restriction and dietary quality. No single ideal ratio of CHO, fat or protein; CHO intake emphasizing nutrient-dense high fiber, including vegetables, legumes, fruits, whole grains, dairy

*AHA, American Heart Association; ADA, American Diabetes Association; ESC, European Society of Cardiology; EASD, European Association for the Study of Diabetes; CHO, carbohydrate; T2D, type 2 diabetes; SFA, saturated fatty acids; USFA, unsaturated fatty acids; en%, percentage of total energy*.

The contribution of bovine milk and its myriad of products to cardiometabolic health is an area of active and still developing research. Large cohort studies, systematic reviews and meta-analyses now point to a neutral or even a protective inverse relationship of dairy with CVD and T2D, but long-term RCTs are as yet lacking. Such long-term well-controlled studies are required in order to build the cause and effect evidence base that is necessary in order for consensus in this long controversial area of nutritional science to finally be reached.

## Author Contributions

The author confirms being the sole contributor of this work and has approved it for publication.

## Conflict of Interest

SP declares that this manuscript was drafted in the absence of any commercial or financial relationships that could be construed as a potential conflict of interest. SP previously held the Fonterra Chair of Human Nutrition at the University of Auckland, New Zealand (2012-2018).
